# Lurasidone Sub-Chronically Activates Serotonergic Transmission via Desensitization of 5-HT1A and 5-HT7 Receptors in Dorsal Raphe Nucleus

**DOI:** 10.3390/ph12040149

**Published:** 2019-10-06

**Authors:** Motohiro Okada, Kouji Fukuyama, Ruri Okubo, Takashi Shiroyama, Yuto Ueda

**Affiliations:** Department of Neuropsychiatry, Division of Neuroscience, Graduate School of Medicine, Mie University, Tsu 514-8507, Japan; k-fukuyama@clin.medic.mie-u.ac.jp (K.F.); ddduck0602@gmail.com (R.O.); takashi@clin.medic.mie-u.ac.jp (T.S.); uedayuto@gmail.com (Y.U.)

**Keywords:** Lurasidone, serotonin, GABA, microdialysis, schizophrenia, mood disorder

## Abstract

Lurasidone is an atypical mood-stabilizing antipsychotic agent with unique receptor-binding profile, including 5-HT7 receptor (5-HT7R) antagonism. Effects of 5-HT7R antagonism on transmitter systems of schizophrenia and mood disorders, however, have not been well clarified. Thus, this study examined the mechanisms underlying the clinical effects of lurasidone by measuring mesocortical serotonergic transmission. Following systemic and local administrations of lurasidone, MK801 and 5-HT receptor modulators, we determined releases of 5-HT in dorsal raphe nucleus (DRN), mediodorsal thalamic nucleus (MDTN) and medial prefrontal cortex (mPFC) and γ-aminobutyric acid (GABA) in DRN using multiprobe microdialysis with ultra-high-performance liquid chromatography (UHPLC). Serotonergic and GABAergic neurons in the DRN are predominantly regulated by inhibitory 5-HT1A receptor (5-HT1AR) and excitatory 5-HT7R, respectively. Lurasidone acutely generates GABAergic disinhibition by 5-HT7R antagonism, but concomitant its 5-HT1AR agonism prevents serotonergic hyperactivation induced by 5-HT7R inhibition. During treatments with 5-HT1AR antagonist in DRN, lurasidone dose-dependently increased 5-HT release in the DRN, MDTN and mPFC. Contrary, lurasidone chronically enhanced serotonergic transmission and GABAergic disinhibition in the DRN by desensitizing both 5-HT1AR and 5-HT7R. These effects of lurasidone acutely prevented MK801-evoked 5-HT release by GABAergic disinhibition via N-methyl-D-aspartate (NMDA)/glutamate receptor (NMDA-R)-mediated inhibition of 5-HT1AR function, but enhanced MK801-induced 5-HT release by desensitizing 5-HT1AR and 5-HT7R. These results indicate that acutely lurasidone fails to affect 5-HT release, but chronically enhances serotonergic transmission by desensitizing both 5-HT1AR and 5-HT7R. These unique properties of lurasidone ameliorate the dysfunctions of NMDA-R and augment antidepressive effects.

## 1. Introduction

Functional abnormalities of monoaminergic and glutamatergic transmissions between frontal cortex and several sub-cortical regions are implicated in cognitive-impairments of schizophrenia and mood disorders [1,2,3,4]. Atypical antipsychotics ameliorate paradoxical dopaminergic dysfunctions, including mesolimbic hyperfunction and mesocortical hypofunction [5,6,7,8,9,10,11,12]. Modulation of serotonergic transmission by 5-HT1A (5-HT1AR) and 5-HT2A (5-HT2AR) receptors in dopaminergic pathophysiology of schizophrenia plays important roles in antipsychotic actions of associated agents [3,8]. Accordingly, selective D2 receptor (D2R) antagonists alone fail to affect frontal dopamine release, whereas combined D2R inhibition with activated inhibitory 5-HT1AR or reduced excitatory 5-HT2AR increased frontal dopamine release [5,6,8,9,10,11,12]. Inhibition of γ-aminobutyric acid (GABA) (GABAergic disinhibition) by inhibitory 5-HT1AR agonism or excitatory 5-HT2AR antagonism is considered mechanisms by which paradoxical actions of some atypical antipsychotics, such as aripiprazole and clozapine, lead to frontal dopamine release [8,12]. 

Recently, the importance of 5-HT7 receptor (5-HT7R) modulation has been a source of scientific discussion in psychiatry and psychopharmacology, because a novel antipsychotic drug lurasidone predominantly blocks 5-HT7R rather than D2R>5-HT2AR and 5-HT1AR [13]. 5-HT7R is the most recently identified member of the 5-HT receptor family, and is an excitatory receptor that binds Gs proteins to activate adenylate cyclase [4]. Lurasidone has been approved for the treatment of schizophrenia in USA, Canada, Australia, and EU [14]. A recent study demonstrated cognition-improving mechanisms of lurasidone, specifically by showing that lurasidone normalizes hyperactivated thalamocortical glutamatergic transmission through blockade of N-methyl-D-aspartate (NMDA)/glutamate receptor (NMDA-R) induced intra-thalamic GABAergic disinhibition via inhibition of 5-HT7R in mediodorsal thalamic nucleus (MDTN) [11]. 

Lurasidone has also been approved for treatment of depressive episodes in bipolar I disorder in USA and Canada [14,15]. Combination 5-HT1AR agonism with 5-HT2AR antagonism contribute to mood stabilizing and antidepressive actions [4]. Preclinical behavioral studies demonstrated that lurasidone had potent effects in rodent models, with likely antipsychotic, antidepressive, and anxiolytic activities in humans [13]. Moreover, subeffective doses of selective 5-HT7R antagonist (2R)-1-[(3-Hydroxyphenyl)sulfonyl]-2-[2-(4-methyl-1-piperidinyl)ethyl]pyrrolidine (SB269970) enhanced the activities of several antidepressants [4,16]. Although the rapid-acting antidepressive action of lurasidone has not been proved in clinical trials, 5-HT7R inhibition is considered novel target of rapid-acting antidepressants. Despite these clinical and preclinical behavioral studies, interactions between 5-HT1AR and 5-HT7R that affect transmission have not been clarified. In previous microdialysis studies, systemic administration of effective doses of lurasidone increased frontal release of dopamine, norepinephrine, and L-glutamate without affecting 5-HT release [11,17], as shown with other atypical antipsychotics such as quetiapine and zotepine [5,6]. Yet the mechanisms behind these discrepancies in effects of lurasidone on 5-HT and catecholamine releases have not been clarified.

The dorsal raphe nucleus (DRN) is the major serotonergic nucleus, and projects to widespread serotonergic terminals, including cortex and thalamus [4]. 5-HT receptor subtypes are also considered regulators of serotonergic transmission that affect serotonergic and GABAergic neurons in the DRN. Accordingly, 5-HT1AR and probably 5-HT7R are expressed in DRN [4,18,19]. Based on these observations, we explored the mechanisms behind antipsychotic and antidepressive actions of lurasidone by determining the effects of lurasidone on serotonergic transmission in the DRN, MDTN and medial prefrontal cortex (mPFC). Specifically, we monitored changes in 5-HT1AR and 5-HT7R activities using multiprobe microdialysis with ultra-high-performance liquid chromatography (UHPLC). 

## 2. Results

The target agents and their application roots in the six studies were summarized in Table 1.

### 2.1. Dose-Dependent Effects of Systemic Lurasidone Administrations on Extracellular Levels of 5-HT in the DRN, MDTN, mPFC, and GABA in the DRN (Study_1)

**Figure 1:**
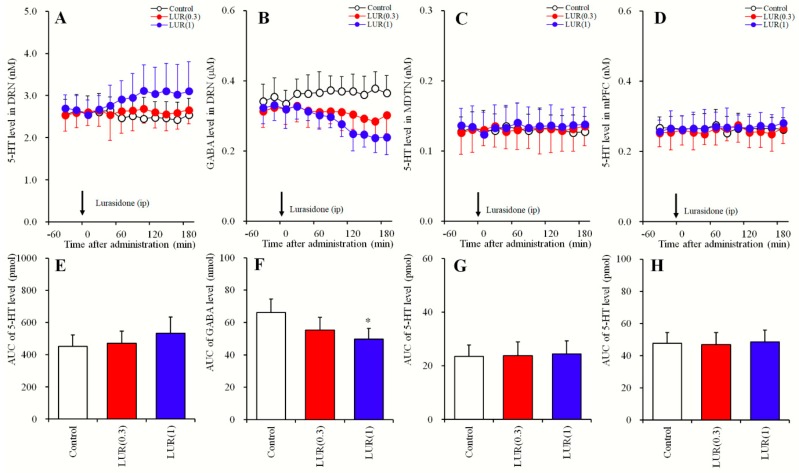
Dose-dependent effects of systemic administration of lurasidone on extracellular levels of 5-HT in the DRN, MDTN, mPFC and γ-aminobutyric acid (GABA) in the DRN (Study_1). (**A**,**C**,**D**) indicate dose-dependent effects of intraperitoneal administration of lurasidone (LUR: 0, 0.3 and 1 mg/kg) on extracellular levels of 5-HT in the DRN, MDTN and mPFC, respectively. (**B**) indicates dose-dependent effects of intraperitoneal administration of lurasidone (LUR: 0, 0.3 and 1 mg/kg) on extracellular GABA level in the DRN. *Y*-axis: Mean ± SD (n = 6) of extracellular levels of 5-HT (nM) and GABA (μM); *X*-axis: Time after administration of lurasidone (min). Arrows indicate intraperitoneal administration of lurasidone. Microdialysis was conducted to measure the releases of 5-HT and GABA. (**E**–**H**) indicate the area under curve (AUC) value of extracellular levels of 5-HT (pmol) and GABA (nmol) after intraperitoneal administration of lurasidone from 20 to 180 min of (**A**–**D**), respectively. Opened, red and blue columns represent the AUC values of 0, 0.3 and 1 mg/kg lurasidone administration, respectively. * P < 0.05; relative to control (lurasidone free) by MANOVA with Tukey’s post hoc test.

Previous studies show that subeffective and effective doses of lurasidone on extracellular transmitter levels are 0.3 and 1 mg/kg, respectively [11,17,20]. According to these studies, study_1 was designed to determine dose-dependent effects of systemic administrations of subeffective (0.3 mg/kg) and effective (1 mg/kg) doses of lurasidone [11,17,20] on extracellular levels of 5-HT in the DRN, MDTN, mPFC, and GABA in the DRN. Perfusion mediums in the DRN, MDTN and mPFC were commenced with MRS. After confirming stabilization of extracellular levels of 5-HT and GABA in perfusates, rats were administered with lurasidone (0, 0.3, or 1 mg/kg) intraperitoneally (Figure 1 and Table 1). 

Systemic administration of lurasidone dose-dependently decreased extracellular GABA levels in the DRN [F_Dose_ (2, 15) = 6.1 (P < 0.05), F_Time_ (8.2, 123.6) = 17.1 (P < 0.05), F_Dose*Time_ (16.5, 123.6) = 11.9 (P < 0.05)] (Figure 1B,F), without affecting those of 5-HT in the DRN, MDTN or mPFC (Figure 1A,C–E,G,H). Extracellular GABA levels in the DRN were decreased by effective doses of lurasidone (1 mg/kg), but not were affected by subeffective (0.3 mg/kg) doses (Figure 1B,F).

### 2.2. Dose-Dependent Effects of Systemic Lurasidone Administration on Extracellular Levels of 5-HT in the DRN, MDTN, mPFC and GABA in the DRN Following Inhibition of 5-HT1AR in the DRN (Study_2)

**Figure 2:**
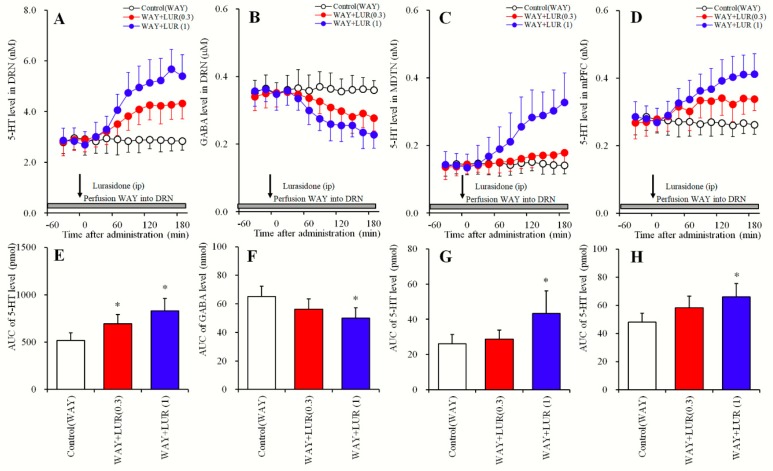
Dose-dependent effects of systemic administration of lurasidone on extracellular levels of 5-HT in the DRN, MDTN, mPFC and GABA in the DRN during 5-HT1AR inhibition in the DRN (Study_2). (**A**,**C**,**D**) indicate dose-dependent effects of systemic administration of lurasidone (LUR: 0, 0.3 and 1 mg/kg) during perfusion with 10 μM N-[2-[4-(2-Methoxyphenyl)-1- piperazinyl]ethyl]-N-2-pyridinylcyclohexanecarboxamide (WAY100635: WAY) into the DRN on extracellular levels of 5-HT in the DRN, MDTN and mPFC, respectively. (**B**) indicates dose-dependent effects of systemic administration of lurasidone (0, 0.3 and 1 mg/kg) during perfusion with 10 μM WAY100635 into the DRN on extracellular GABA level in the DRN. *Y*-axis: Mean ± SD (n = 6) of extracellular levels of 5-HT (nM) and GABA (μM); *X*-axis: Time after administration of lurasidone (min). Arrows indicate intraperitoneal administration of lurasidone, and gray bars indicate the perfusion with 10 μM WAY100635. Microdialysis was conducted to measure the releases of 5-HT and GABA. (**E**–**H**) indicate the AUC value of extracellular levels of 5-HT (pmol) and GABA (nmol) after systemic administration of lurasidone from 20 to 180 min of (**A**–**D**), respectively. Opened, red and blue columns represent the AUC values of 0, 0.3 and 1 mg/kg lurasidone administration, respectively. * P < 0.05; relative to control (lurasidone free) by MANOVA with Tukey’s post hoc test.

In the DRN, 5-HT1AR is a major autoregulation inhibitory receptor against serotonergic neurons in the DRN [18,22]. Study_2 was designed to determine dose-dependent effects of systemic lurasidone administrations at 0, 0.3, or 1 mg/kg, and distinguish these from the effects of 5-HT1AR on extracellular levels of 5-HT in the DRN, MDTN, mPFC, and GABA in the DRN. Perfusate in the DRN was commenced with MRS containing 10 μM N-[2-[4-(2-Methoxyphenyl)- 1-piperazinyl]ethyl]-N-2-pyridinylcyclohexanecarboxamide (WAY100635) (5-HT1AR antagonist), and perfusions in the MDTN and the mPFC were commenced with MRS. After confirming stabilization of extracellular levels of 5-HT and GABA in perfusates, rats were administered lurasidone (0, 0.3, or 1 mg/kg) intraperitoneally (Figure 2 and Table 1).

During inhibition of 5-HT1AR in the DRN by perfusions with WAY100635 (10 μM) into the DRN, systemic lurasidone administrations dose-dependently decreased extracellular GABA levels in the DRN [F_Dose_ (2,15) = 5.4 (P < 0.05), F_Time_ (9,135) = 73.5 (P < 0.05), F_Dose*Time_ (18,135) = 23.1 (P < 0.05)] (Figure 2B,F). Subeffective doses of lurasidone (0.3 mg/kg), however, did not affect extracellular GABA levels in the DRN, whereas effective doses (1 mg/kg) decreased GABA levels (Figure 2B,F).

In contrast with GABA, extracellular levels of 5-HT in the DRN [F_Dose_ (2,15) = 11.2 (P < 0.05), F_Time_ (5.7, 84.7) = 133.9 (P < 0.05), F_Dose*Time_ (11.3, 84.8) = 44.5 (P < 0.05)], MDTN [F_Dose_ (2, 15) = 6.3 (P < 0.05), F_Time_ (5.1, 76.5) = 57.3 (P < 0.05), F_Dose*Time_ (10.2, 76.5) = 33.8 (P < 0.05)] and mPFC [F_Dose_ (2, 15) = 6.0 (P < 0.05), F_Time_ (9, 135) = 41.9 (P < 0.05), F_Dose*Time_ (18, 135) = 21.2 (P < 0.05)] were dose-dependently increased by systemic administration of lurasidone (Figure 2A,C–E,G,H). Extracellular 5-HT levels in the DRN were also increased by subeffective (0.3 mg/kg) and effective (1 mg/kg) doses of lurasidone (Figure 2A,E). However, extracellular levels of 5-HT in the MDTN and mPFC were increased by effective doses but were not affected by subeffective doses (Figure 2C,D,G,H). 

### 2.3. Concentration-Dependent Effects of Local Lurasidone Administrations into the DRN on Extracellular Levels of 5-HT in the DRN, MDTN, mPFC and GABA in the DRN (Study_3)

Both study_1 and study_2 indicate that systemic administration of lurasidone dose-dependently increased 5-HT release in the DRN without affecting those in the MDTN or mPFC; however, under the 5-HT1AR inhibition in the DRN, systemic administration of lurasidone increased 5-HT release in all three regions with reduced GABA release in the DRN. To clarify the mechanisms of these discrepant effects of systemic administration of lurasidone under the conditions between 5-HT1AR functional and inhibition, the present study determined the DRN regional specific action of lurasidone, 5-HT1AR and 5-HT7R on 5-HT release and GABA in the DRN.

**Figure 3:**
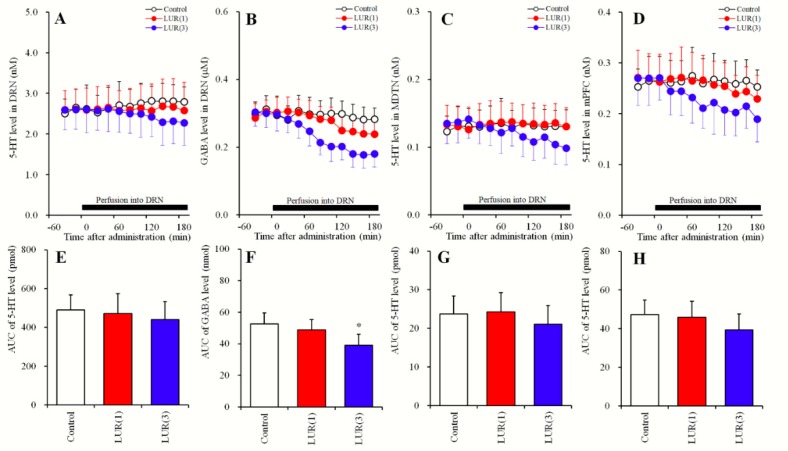
Concentration-dependent effects of local administration of lurasidone into the DRN on extracellular levels of 5-HT in the DRN, MDTN, mPFC and GABA in the DRN (Study_3-1). (**A**,**C**,**D**) indicate concentration-dependent effects of perfusion with lurasidone (LUR: 0, 1 and 3 μM) on extracellular levels of 5-HT in the DRN, MDTN and mPFC, respectively. (**B**) indicates concentration-dependent effects of perfusion with lurasidone (LUR: 0, 1 and 3 μM) on extracellular GABA levels in the DRN. *Y*-axis: Mean ± SD (n = 6) of extracellular levels of 5-HT (nM) and GABA (μM); *X*-axis: Time after administration of lurasidone (min). Closed bars indicate perfusion with lurasidone (LUR: 1 or 3 μM). Microdialysis was conducted to measure the releases of 5-HT and GABA. (**E**–**H**) indicate the AUC value of extracellular levels of 5-HT (pmol) and GABA (nmol) during perfusion with lurasidone from 20 to 180 min of (**A**–**D**), respectively. Opened, red and blue columns represent the AUC values of perfusion with 0, 1 and 3 μM lurasidone, respectively. * P < 0.05; relative to control (lurasidone free) by MANOVA with Tukey’s post hoc test.

**Figure 4:**
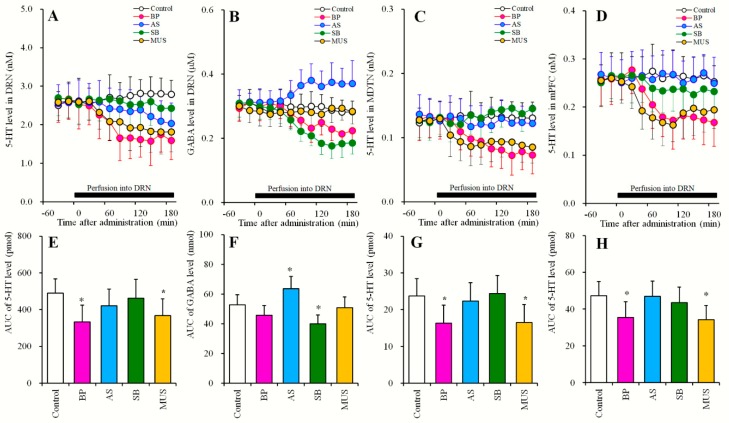
Effects of local administration of BP554, AS19, SB269970 and muscimol into the DRN on extracellular levels of 5-HT in the DRN, MDTN, mPFC, and GABA in the DRN (Study_3-2). (**A**,**C**,**D**) indicate effects of perfusion with 1-[3-(3,4-Methylenedioxyphenoxy) propyl]-4-phenyl- piperazine (BP: 50 μM), (2S)-(+)-5-(1,3,5- Trimethylpyrazol-4-yl)-2- (dimethylamino)tetralin (AS: 1 μM), (2R)-1-[(3-Hydroxyphenyl)sulfonyl]- 2-[2-(4-methyl-1- piperidinyl)ethyl]pyrrolidine (SB: 10 μM) and muscimol (MUS: 1 μM) on extracellular levels of 5-HT in the DRN, MDTN and mPFC, respectively. (**B**) indicates effects of perfusion with BP554, AS19, SB269970 and muscimol into the DRN on extracellular GABA levels in the DRN. *Y*-axis: Mean ± SD (n = 6) of extracellular levels of 5-HT (nM) and GABA (μM); *X*-axis: Time after administration of target agents (min). Closed bars indicate perfusion with target agents into the DRN. Microdialysis was conducted to measure the releases of 5-HT and GABA. (**E**–**H**) indicate the AUC value of extracellular levels of 5-HT (pmol) and GABA (nmol) during perfusion with target agents from 20 to 180 min of (**A**–**D**), respectively. Opened, pink, light blue, green and orange columns represent the AUC values of perfusion with control, BP554, AS19, SB269970 and muscimol, respectively. * P < 0.05; relative to control by MANOVA with Tukey’s post hoc test.

Our previous studies showed that local administration of 3 μM lurasidone into the insular cortex increased regional extracellular levels of dopamine and norepinephrine, whereas lower than 1 μM had no effects [11]. Therefore, study_3-1 was designed to determine the concentration- dependent effects of local lurasidone administrations in the DRN, and study_3-2 was designed to determine the effects of 5-HT1AR, 5-HT7R, and GABA_A_ receptors (GABA_A_-R) in the DRN on extracellular levels of 5-HT in the DRN, MDTN, mPFC, and GABA in the DRN. In study_3, perfusate in the DRN, MDTN and mPFC were commenced with MRS. After confirming stabilization of extracellular 5-HT and GABA levels in perfusates, perfusates in the DRN were switched to MRS containing lurasidone (1 or 3 μM), 5-HT1AR agonist, 1-[3-(3,4-Methylene- dioxyphenoxy)- propyl]-4-phenyl-piperazine (BP554: 50 μM), 5-HT7R agonist, (2S)-(+)-5-(1,3,5- Trimethylpyrazol-4-yl)-2-(dimethylamino)tetralin (AS19: 1 μM), 5-HT7R antagonist, (2R)-1-[(3- Hydroxy-phenyl)sulfonyl]-2-[2-(4-methyl-1-piperidinyl)ethyl]pyrrolidine (SB269970: 10 μM), or GABA_A_-R agonist, muscimol (1 μM) for 180 min (Figure 3 and Figure 4 and Table 1). The perfusates in the MDTN and mPFC were remained MRS during experiment. 

#### 2.3.1. Concentration-Dependent Effects of Local Lurasidone Administrations into the DRN on Extracellular Levels of 5-HT in the DRN, MDTN, mPFC and GABA in the DRN (Study_3-1)

Perfusions with lurasidone (0, 1 and 3 μM) into the DRN did not affect extracellular levels of 5-HT in the DRN, MDTN or mPFC (Figure 3A,C–E,G,H). Perfusion with lurasidone into the DRN decreased regional extracellular GABA levels [F_Level_ (2, 15) = 4.9 (P < 0.05), F_Time_ (5.7, 57.2) = 68.7 (P < 0.05), F_Level*Time_ (11.5, 85.9) = 17.7 (P < 0.05)] (Figure 3B,F). Moreover, perfusion with 3 μM lurasidone decreased extracellular GABA levels in the DRN, whereas 1 μM lurasidone perfusions had no effects (Figure 3B,F). 

#### 2.3.2. Effects of Local Administrations of BP554, AS19, SB269970 and Muscimol into the DRN on Extracellular Levels of 5-HT in the DRN, MDTN, mPFC, and GABA in the DRN (Study_3-2)

Perfusions with 50 μM BP554 into the DRN decreased extracellular 5-HT level in the DRN [F_BP_ (1,10) = 8.1 (P < 0.05), F_Time_ (9,90) = 12.2 (P < 0.05), F_BP*Time_ (9,90) = 30.6 (P < 0.05)] (Figure 4A,E), MDTN [F_BP_ (1,10) = 5.7 (P < 0.05), F_Time_ (6.4, 63.9) = 20.6 (P < 0.05), F_BP*Time_ (6.4, 63.9) = 22.7 (P < 0.05)] (Figure 4C,G) and mPFC [F_BP_ (1,10) = 5.1 (P < 0.05), F_Time_ (7.1, 70.6) = 26.2 (P < 0.05), F_BP*Time_ (7.1, 70.6) = 25.8 (P < 0.05)] (Figure 4D,H) without affecting extracellular GABA levels in the DRN (Figure 4B,F). Similar to BP554, perfusions with 1 μM muscimol into the DRN decreased extracellular 5-HT levels in the DRN [F_MUS_ (1, 10) = 5.1 (P < 0.05), F_Time_ (9, 90) = 7.7 (P < 0.05), F_MUS*Time_ (9,90) = 28.9 (P < 0.05)] (Figure 4AE), MDTN [F_MUS_ (1, 10) = 5.6 (P < 0.05), F_Time_ (7.1, 70.6) = 7.1 (P < 0.05), F_MUS*Time_ (7.1, 70.6) = 9.9 (P < 0.05)] (Figure 4C,G) and mPFC [F_MUS_ (1,10) = 7.2 (P < 0.05), F_Time_ (9, 90) = 9.7 (P < 0.05), F_MUS*Time_ (9, 90) = 12.6 (P < 0.05)] (Figure 4D,H) without affecting extracellular GABA levels in the DRN (Figure 4B,F). 

Perfusions with 1 μM AS19 into the DRN increased extracellular GABA levels in the DRN [F_AS_ (1, 10) = 5.3 (P < 0.05), F_Time_ (9, 90) = 6.1 (P < 0.05), F_AS*Time_ (9, 90) = 8.8 (P < 0.05)] (Figure 4B,F) without affecting 5-HT levels in the DRN, MDTN or mPFC (Figure 4A,C–E,G,H). Perfusions with 10 μM SB269970 into the DRN decreased extracellular GABA levels in the DRN [F_SB_ (1, 10) = 8.9 (P < 0.05), F_Time_ (5.8, 57.7) = 51.4 (P < 0.05), F_SB*Time_ (5.8, 57.7) = 38.9 (P < 0.05)] (Figure 4BF) without affecting 5-HT levels in the DRN, MDTN or mPFC (Figure 4A,C–E,G,H). 

The results of Study_3 indicate that in the DRN, serotonergic transmission is regulated by inhibitory 5-HT1AR and GABA_A_-R rather than by 5-HT7R, whereas GABAergic transmission is regulated by 5-HT7R rather than 5-HT1AR and GABA_A_-R. 

### 2.4. Effects of Local Administrations of AS19, SB269970 and Lurasidone into the DRN on Extracellular Levels of 5-HT in the DRN, MDTN, mPFC, and GABA in the DRN Following Inhibition of 5-HT1AR in the DRN (Study_4)

**Figure 5:**
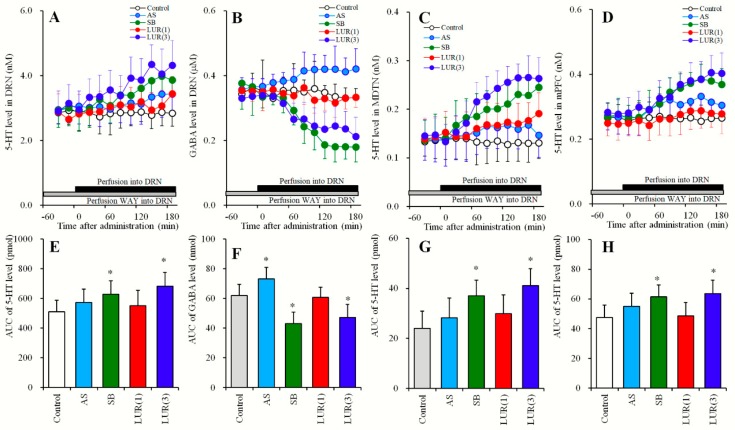
Effects of local administration of AS19, SB269970 and lurasidone into the DRN on extracellular levels of 5-HT in the DRN, MDTN, mPFC, and GABA in the DRN under the inhibition of 5-HT1AR in the DRN (Study_4). (**A**,**C**,**D**) indicate effects of perfusion with AS19 (AS: 1 μM), SB269970 (SB: 10 μM) and lurasidone (LUR: 1 and 3 μM) during perfusion with 10 μM WAY100635 into the DRN on extracellular levels of 5-HT in the DRN, MDTN and mPFC, respectively. (**B**) indicates effects of perfusion with AS19, SB269970 and lurasidone into the DRN during perfusion with WAY100635 on extracellular GABA level in the DRN. *Y*-axis: Mean ± SD (n = 6) of extracellular levels of 5-HT (nM) and GABA (μM); *X*-axis: Time after administration of target agents (min). Closed bars indicate perfusion with target agents, and gray bars indicate perfusion with WAY100635. Microdialysis was conducted to measure the releases of 5-HT and GABA. (**E**–**H**) indicate the AUC value of extracellular levels of 5-HT (pmol) and GABA (nmol) during perfusion with target agents from 20 to 180 min of (**A**–**D**), respectively. Opened, light blue, green, red and blue columns represent the AUC values of perfusion with control, AS19, SB269970, 1 μM and 3 μM lurasidone, respectively. * P < 0.05; relative to control by MANOVA with Tukey’s post hoc test.

In studies_1~3, lurasidone did not affect 5-HT release in the presence of functional 5-HT1AR in the DRN, but when 5-HT1AR was blocked, lurasidone administration enhanced 5-HT release. Study_4 was designed to determine concentration-dependent effects of local administration of lurasidone in the DRN, and the effects of 5-HT7R in the DRN on extracellular levels of 5-HT in the DRN, MDTN, mPFC, and GABA in the DRN, following inhibition of 5-HT1AR in the DRN. Perfusate in the DRN were commenced with MRS containing 10 μM WAY100635, and perfusions in the MDTN and mPFC were commenced with MRS. After confirming that 5-HT and GABA levels were stable in perfusates, the perfusate in the DRN was switched to MRS containing WAY100635 plus lurasidone (0, 1 or 3 μM), AS19 (1 μM; 5-HT7R agonist), or SB269970 (10 μM; 5-HT7R antagonist) for 180 min (Figure 5 and Table 1). The perfusates in the MDTN and mPFC were continued MRS during this study.

Perfusions with 1 μM AS19 into the DRN did not affect extracellular levels of 5-HT in the DRN, MDTN or mPFC, but decreased GABA level in the DRN [F_AS_ (1, 10) = 5.4 (P < 0.05), F_Time_ (3.8, 37.5) = 2.5 (P > 0.05), F_AS*Time_ (3.8, 37.5) = 3.5 (P < 0.05)] under 5-HT1AR blockade in the DRN (Figure 5B,F). Under the same conditions, perfusion with 10 μM SB269970 into the DRN increased extracellular levels of 5-HT in the DRN [F_SB_ (1, 10) = 5.1 (P < 0.05), F_Time_ (7.3, 72.5) = 15.7 (P < 0.05), F_SB*Time_ (7.3, 72.5) = 16.7 (P < 0.05)], MDTN [F_SB_ (1, 10) = 9.5 (P < 0.05), F_Time_ (9, 90) = 13.0 (P < 0.05), F_SB*Time_ (9, 90) = 18.6 (P < 0.05)] and mPFC [F_SB_ (1, 10) = 7.3 (P < 0.05), F_Time_ (4.9, 49.3) = 23.1 (P < 0.05), F_SB*Time_ (4.9, 49.3) = 25.8 (P < 0.05)], and decreased GABA level in the DRN [F_SB_ (1, 10) = 13.8 (P < 0.05), F_Time_ (2.8, 27.5) = 35.6 (P < 0.05), F_SB*Time_ (2.8, 27.5) = 28.7 (P < 0.05)] (Figure 5).

Similar to SB269970, when 5-HT1AR was blocked in the DRN, perfusions with lurasidone into the DRN increased extracellular levels of 5-HT in the DRN [F_LUR_ (2, 15) = 4.5 (P < 0.05), F_Time_ (5.8, 87.6) = 22.8 (P < 0.05), F_LUR*Time_ (11.7, 87.6) = 12.6 (P < 0.05)], MDTN [F_LUR_ (2, 15) = 7.7 (P < 0.05), F_Time_ (6.2, 93.7) = 42.9 (P < 0.05), F_LUR*Time_ (12.5, 93.7) = 25.1 (P < 0.05)] and mPFC [F_LUR_ (2, 15) = 5.7 (P < 0.05), F_Time_ (9, 135) = 24.2 (P < 0.05), F_LUR*Time_ (18, 135) = 12.2 (P < 0.05)], and decreased GABA level in the DRN [F_LUR_ (2, 15) = 5.8 (P < 0.05), F_Time_ (4.0, 59.9) = 15.7 (P < 0.05), F_LUR*Time_ (8.0, 59.9) = 7.9 (P < 0.05)] (Figure 5). 

### 2.5. Effects of Local Administrations of BP554, SB269970, and Muscimol in the DRN on Changes in Extracellular Levels of 5-HT in the DRN, MDTN, mPFC and GABA in the DRN (Study_5)

**Figure 6:**
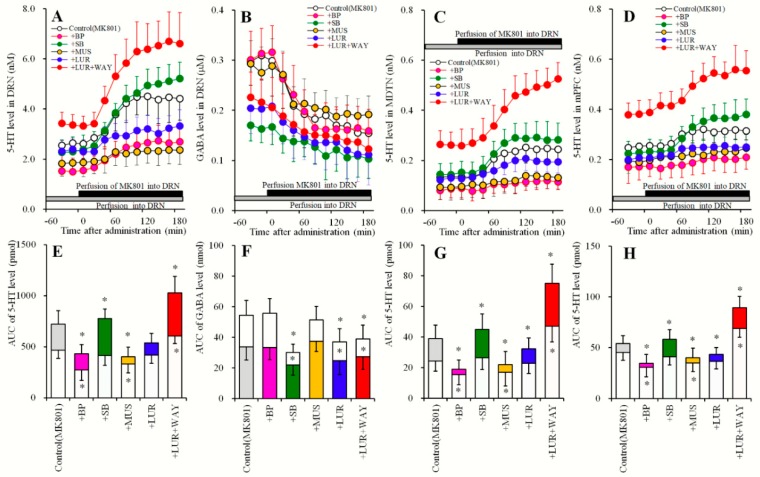
Effects of local administration of BP554, SB269970 and muscimol into the DRN on MK801-evoked 5-HT release in the DRN, MDTN, mPFC and MK801-induced GABA reduction in the DRN (Study_5). (**A**,**C**,**D**) indicate effects of perfusion with BP554 (BP: 10 μM), SB269970 (SB: 10 μM) and muscimol (MUS: 1 μM) on 5 μM MK801-evoked 5-HT release in the DRN, MDTN and mPFC, respectively. (**B**) indicates effects of perfusion with BP554, SB269970 and muscimol into the DRN on MK801-induced GABA reduction in the DRN. *Y*-axis: Mean ± SD (n = 6) of extracellular levels of 5-HT (nM) and GABA (μM); *X*-axis: Time after administration of MK801 (min). Closed bars indicate perfusion with 5 μM MK801, and gray bars indicate perfusion with target agents. Microdialysis was conducted to measure the releases of 5-HT and GABA. (**E**–**H**) indicate the AUC value of extracellular levels of 5-HT (pmol) and GABA (nmol) during perfusion with MK801 from 20 to 180 min of respective (**A**–**D**). Opened, gray, pink, light blue, green and orange columns represent the AUC values of before and after perfusion with MK801 during perfusion of control, BP554, SB269970 and muscimol, respectively. * P < 0.05; relative to control by MANOVA with Tukey’s post hoc test.

Study_5 was designed to determine the effects of local administrations of agents of 5-HT1AR, 5-HT7R, GABA_A_-R and lurasidone into the DRN on MK801-induced 5-HT release and MK801-induced GABA reductions (Figure 6). After study_3 or Study_4, perfusion media in the DRN were switched to the same MRS containing with 5 μM MK801 for 180 min (Figure 6 and Table 1). The perfusates in the MDTN and mPFC were continued MRS during experiment.

Local administration of 5 μM MK801 (NMDA-R antagonist) into the DRN increased extracellular 5-HT levels in the DRN, MDTN and mPFC (MK801-evoked 5-HT release) and decreased extracellular GABA levels in the DRN (MK801-induced GABA reduction) (Figure 6). MK801-induced 5-HT release in the DRN was inhibited by perfusions with 50 μM BP554 [F_BP_ (1, 10) = 9.2 (P < 0.05), F_Time_ (5.5, 54.8) = 75.1 (P < 0.05), F_BP*Time_ (5.5, 54.8) = 5.3 (P < 0.05)], and by perfusions with 1 μM muscimol [F_MUS_ (1, 10) = 22.8 (P < 0.05), F_Time_ (5.0, 50.0) = 47.6 (P < 0.05), F_MUS*Time_ (5.0, 50.0) = 16.9 (P < 0.05)] into the DRN, but was enhanced by perfusion with 10 μM SB269970 [F_SB_ (1, 10) = 8.4 (P < 0.05), F_Time_ (7.3, 73.4) = 130.3 (P < 0.05), F_SB*Time_ (7.4, 73.4) = 5.7 (P < 0.05)] (Figure 6A,E). MK801-induced 5-HT release in the MDTN was inhibited by perfusions with 50 μM BP554 [F_BP_ (1, 10) = 19.4 (P < 0.05), F_Time_ (9, 90) = 75.8 (P < 0.05), F_BP*Time_ (9, 90) = 5.3 (P < 0.05)] and 1 μM muscimol into the DRN [F_MUS_ (1, 10) = 10.3 (P < 0.05), F_Time_ (6.2, 62.9) = 59.2 (P < 0.05), F_MUS*Time_ (6.2, 61.9) = 6.6 (P < 0.05)]. MK801-induced 5-HT release was, however, enhanced by perfusion with 10 μM SB269970 into the DRN [F_SB_ (1, 10) = 6.2 (P < 0.05), F_Time_ (7.5, 74.8) = 145.2 (P < 0.05), F_SB*Time_ (7.5, 74.8) = 2.5 (P < 0.05)] (Figure 6C,G). MK801-induced 5-HT release in the mPFC was inhibited by perfusions with 50 μM BP554 [F_BP_ (1, 10) = 15.9 (P < 0.05), F_Time_ (6.7, 67.2) = 25.9 (P < 0.05), F_BP*Time_ (6.7, 67.2) = 3.7 (P < 0.05)] and by perfusions with 1 μM muscimol [F_MUS_ (1, 10) = 7.6 (P < 0.05), F_Time_ (9, 90) = 21.6 (P < 0.05), F_MUS*Time_ (9, 90) = 4.9 (P < 0.05)] into the DRN, but was enhanced by perfusions with 10 μM SB269970 [F_SB_ (1, 10) = 34.5 (P < 0.05), F_Time_ (7.9, 79.2) = 93.8 (P < 0.05), F_SB*Time_ (7.9, 79.2) = 16.3 (P < 0.05)] (Figure 6D,H).

In contrast to 5-HT, MK801-induced GABA reduction in the DRN were inhibited by DRN perfusions containing 10 μM SB269970 [F_SB_ (1, 10) = 8.8 (P < 0.05), F_Time_ (6.0, 60.0) = 54.4 (P < 0.05), F_SB*Time_ (6.0, 60.0) = 11.7 (P < 0.05)], but were not affected by DRN perfusion with BP554 or muscimol (Figure 6B,F). 

MK801-induced 5-HT release in the DRN [F_LUR_ (2, 15) = 26.8 (P < 0.05), F_Time_ (4.9, 73.2) = 122.9 (P < 0.05), F_LUR*Time_ (9.8, 73.2) = 15.8 (P < 0.05)], MDTN [F_LUR_ (2, 15) = 65.8 (P < 0.05), F_Time_ (7.3, 108.7) = 230.0 (P < 0.05), F_LUR*Time_ (33.9, 108.7) = 33.9 (P < 0.05)] and mPFC [F_LUR_ (2, 15) = 44.5 (P < 0.05), F_Time_ (4.2, 63.3) = 69.5 (P < 0.05), F_LUR*Time_ (8.5, 63.3) = 13.3 (P < 0.05)] were inhibited and enhanced by perfusions with lurasidone (3 μM) and lurasidone (3 μM) plus WAY100635 (10 μM), respectively (Figure 6A,C–E,G,H). MK801-induced GABA reduction in the DRN were inhibited by perfusions with lurasidone (3 μM) and lurasidone (3 μM) plus WAY100635 (10 μM) [F_LUR_ (2, 15) = 35.8 (P < 0.05), F_Time_ (9, 135) = 79.2 (P < 0.05), F_LUR*Time_ (9, 135) = 3.4 (P < 0.05)] (Figure 6B,F). 

### 2.6. Effects of Local Administration of MK801, AS19 and BP554 in the DRN on 5-HT Release in the DRN, MDTN, mPFC, and GABA in the DRN After Sub-Chronic Administration of Lurasidone (Study_6)

**Figure 7:**
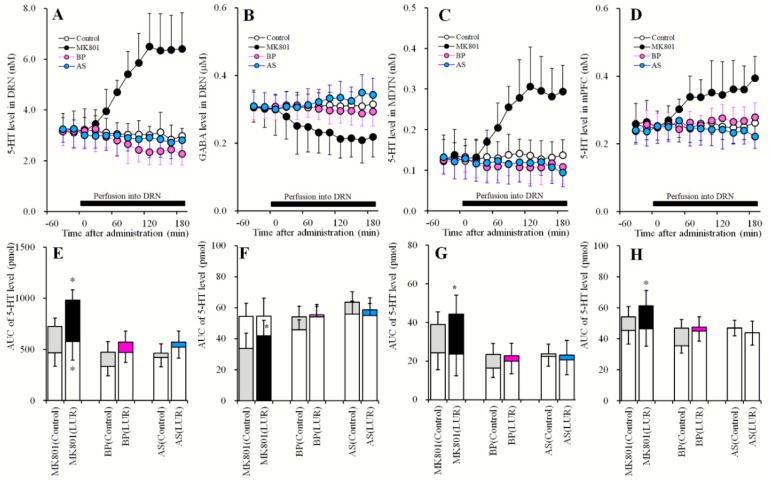
Effects of local administration of MK801, BP554 and AS19 into the DRN on 5-HT releases in the DRN, MDTN, mPFC, and GABA in the DRN, after sub-chronic administration of lurasidone (Study_6). (**A**,**C**,**D**) indicate effects of perfusion with MK801 (5 μM), BP554 (BP: 50 μM) and AS19 (AS: 1 μM) into the DRN on extracellular levels of 5-HT in the DRN, MDTN and mPFC, respectively, after the sub-chronic administration of effective dose of lurasidone (3 mg/kg/day for 7 days). (**B**) indicates effects of perfusion with MK801, BP554 and AS19 into the DRN on extracellular GABA level in the DRN, after sub-chronic administration of effective dose of lurasidone. *Y*-axis: Mean ± SD (n = 6) of extracellular levels of 5-HT (nM) and GABA (μM); *X*-axis: Time after administration of target agents (min). Closed bars indicate perfusion with target agents into the DRN. Microdialysis was conducted to measure the releases of 5-HT and GABA. (**E**–**H**) indicate the AUC value of extracellular levels of 5-HT (pmol) and GABA (nmol) during perfusion with MK801, BP554 or AS19 from 20 to 180 min. Opened columns indicate the AUC values of pre-perfusion of target agents in Figure 4 and Figure 7. Gray columns indicate the AUC values of during perfusion with target agents (MK801, BP554 and AS19) in Figure 4. Closed, pink and light blue columns indicate AUC values of during perfusion with target agents (MK801, BP554 and AS19) in (**A**–**D**), respectively. * P < 0.05; relative to control (without sub-chronic lurasidone administration) by MANOVA with Tukey’s post hoc test.

Study_6 was designed to determine the effects of systemically sub-chronic administration of effective does of lurasidone (3 mg/kg/day for 7 days) [11] on 5 μM MK801-, 50 μM BP554- and 1 μM AS19-induced release of 5-HT and GABA (Figure 7 and Table 1). After sub-chronic systemic administration of effective doses of lurasidone (3 mg/kg/day) for 7 days using an osmotic pumps (2ML_1; Alzet, Cupertino, CA) implanted subcutaneously in the dorsal region (N = 24), microdialysis probes were implanted. Perfusion experiments were initiated at 18 h after recovery from isoflurane anesthesia (36 h after withdraw from sub-chronic lurasidone treatments). A previous study of pharmacokinetics in rats reported a half-life (T_1/2_) of lurasidone of 6–9 h [23]. According to this evidence, we started perfusions at 36 h after withdraw from sub-chronic lurasidone treatments, and avoided the effects of residual lurasidone. Perfusions into the DRN, MDTN and mPFC were started with MRS. After confirming that 5-HT and GABA levels were stable in perfusates, perfusates in the DRN were switched to MRS containing 5 μM MK801, 50 μM BP554, or 1 μM AS19 for 180 min (Figure 7). The perfusates in the MDTN and mPFC were remained MRS during experiment.

The inhibitory effects of perfusions with 50 μM BP554 into the DRN on extracellular 5-HT levels in the DRN, MDTN and mPFC (Figure 4) were abolished by sub-chronic treatments with effective doses of lurasidone (3 mg/kg/day for 7 days; Figure 7A,C–E,G,H). Similar to BP554, the stimulatory effects of perfusion with 1 μM AS19 into the DRN on regional extracellular GABA levels (Figure 4B,F) were abolished by sub-chronic treatments with effective doses of lurasidone (Figure 7B,F). Thus, sub-chronic administration of effective doses of lurasidone may desensitize or downregulate 5-HT1AR and 5-HT7R. 

Contrary to the effects of BP554 and AS19, sub-chronic administration of effective doses of lurasidone enhanced 5 μM MK801-induced 5-HT release in the DRN [F_administration_ (1, 10) = 7.7 (P < 0.05), F_Time_ (4.3, 42.5) = 76.6 (P < 0.05), F_administration*Time_ (4.3, 42.5) = 6.2 (P < 0.05)], MDTN [F_administration_ (1, 10) = 16.5 (P < 0.05), F_Time_ (4.5, 44.8) = 70.9 (P < 0.05), F_administration*Time_ (4.5, 44.8) = 2.7 (P < 0.05)] and mPFC [F_administration_ (1, 10) = 12.3 (P < 0.05), F_Time_ (4.9, 48.7) = 24.3 (P < 0.05), F_administration*Time_ (4.9, 48.7) = 2.1 (P > 0.05)]. These doses of lurasidone also enhanced MK801-induced GABA reduction in the DRN [F_administration_ (1, 10) = 35.5 (P < 0.05), F_Time_ (9, 90) = 64.1 (P < 0.05), F_administration*Time_ (9, 90) = 3.9 (P < 0.05)] (Figure 7). 

## 3. Discussion

### 3.1. Regulatory Mechanisms of Serotonergic Transmission Associated with the DRN

**Figure 8:**
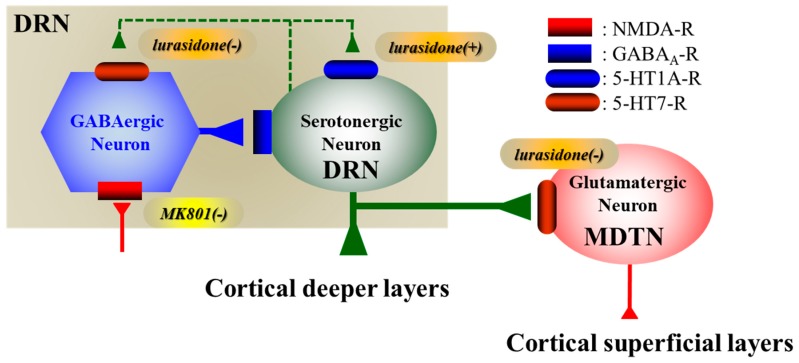
Proposed hypothesis for the extended neural circuitry involved in the DRN, MDTN and mPFC. Our proposed hypothesis for the extended neural circuitry involved in the transmissions of DRN-MDTN and the DRN-mPFC pathways. Serotonergic neurons in the DRN (green circle), which receives intra-DRN GABAergic inhibition, project terminals to the MDTN and deeper layers in the mPFC. GABAergic neurons in the DRN are predominantly regulated by excitatory 5-HT7R and NMDA-R rather than inhibitory 5-HT1AR. In contrast, serotonergic neurons in the DRN are regulated by inhibitory 5-HT1AR rather than excitatory 5-HT7R and NMDA-R. MK801 inhibits NMDA-R on the GABAergic neurons resulting in increase in 5-HT releases in the DRN, MDTN and mPFC; however, lurasidone acutely also enhances GABAergic disinhibition via its 5-HT7R antagonism but inhibits serotonergic neuronal activity via its 5-HT1AR agonism in the DRN. Contrary to acute treatment, lurasidone chronically, enhances 5-HT release via desensitization of 5-HT1AR and 5-HT7R in the DRN.

Serotonergic modulations, including 5-HT release, receptors, transporters, and enzymes, have well established roles in the pathophysiology of schizophrenia, mood disorders, and anxiety disorders [1,2,3,4,11,18,22,24,25]. The DRN is a major serotonergic nucleus that projects terminals to various brain regions, including the mPFC and MDTN [26,27] (Figure 8). Therefore, activation of serotonergic transmission from the DRN is considered fundamental to the pathophysiology of cognitive impairments and mood disturbances [1,2,3,4,11,18,22,24,25]. 

The DRN comprises heterogeneous serotonergic, GABAergic, and glutamatergic neurons [4,19], which may correspond with a variety of physiological and pathological serotonergic functions. This heterogeneous organization complicates the understanding of regulatory mechanisms of serotonergic transmission in the DRN. Therefore, understanding of such properties of GABAergic and serotonergic neuronal activities in the DRN may reveal various functions of the DRN. Our hypothesis for neural circuitry in the interaction between serotonergic and GABAergic transmissions in the DRN is represented in Figure 8.

Herein, we demonstrate that 5-HT release in the DRN is regulated by inhibitory 5-HT1AR and GABA_A_-R predominantly in the DRN, as indicated by decreased regional 5-HT release following local perfusions with BP554 (5-HT1AR agonist) and muscimol (GABA_A_-R agonist) into the DRN (Figure 4). Contrary to 5-HT, GABA release in the DRN is regulated more by excitatory 5-HT7R than 5-HT1AR in the DRN, reflecting decreased regional GABA release following local perfusions with SB269970 (5-HT7R antagonist) into the DRN (Figure 4). 

Previous electrophysiological studies show that inhibitory 5-HT1AR and excitatory 5-HT2AR responses in serotonergic neurons were more than 90% and lower than 20% in the DRN, respectively [19]. In contrast with serotonergic neurons, inhibitory and excitatory 5-HT responses in GABAergic neurons were 15% and 80% in the DRN, respectively, likely relating to 5-HT7R excitatory responses [19]. Taken with these reports, our data suggest that 5-HT1AR and 5-HT7R predominantly inhibit serotonergic neurons and excite GABAergic neurons, respectively. 

Feedback inhibition via somatodendritic 5-HT1AR autoreceptor on serotonergic neurons in the DRN plays an important role in the regulation of 5-HT-mediated neuronal activity [18,22,28]. It has been established that GABAergic transmission in the DRN is also an important regulatory mechanism for controlling serotonergic neuronal activity [28]. Therefore, increased 5-HT release in the DRN enhances negative feedback for serotonergic transmission by activating 5-HT1AR, which directly inhibits serotonergic neuronal activity, and 5-HT7R, which generates GABAergic disinhibition. Furthermore, the negative feedback mechanisms between serotonergic and GABAergic interactions are phasic with each other.

### 3.2. 5-HT1AR-Assoicated Mechanisms of Lurasidone 

Systemic and local administrations of lurasidone concentration-dependently decreased GABA release in the DRN, whereas lurasidone did not affect 5-HT release in the DRN, MDTN or mPFC (Figure 1 and Figure 3). Hence, lurasidone generates GABAergic disinhibition in the DRN but does not increase 5-HT release. These paradoxes are probably mediated by the 5-HT1AR agonistic actions of lurasidone, because lurasidone increased 5-HT release during 5-HT1AR blockade by WAY100635 (Figure 2 and Figure 6). In the present study, WAY100635 was used as a relatively selective 5-HT1AR antagonist (Ki = 2.2 nM); however, initial screens performed by the NIMH Psychoactive Drug Screening Program indicated that Ki values of WAY100635 at D4 receptor is 16 nM [29]. Therefore, WAY100635 is a 5-HT1AR antagonist and D4 receptor agonist [29]. Contrary to WAY100635, lurasidone is high affinity antagonist to 5-HT7R (Ki = 0.5 nM) > D2 receptor (Ki = 1.7 nM) > 5-HT2AR (Ki = 2.0 nM) > 5-HT1AR (Ki = 6.8 nM) > D4 receptor (Ki = 29.7 nM) [30,31]. Based on these previous binding studies, we should discuss whether is the stimulatory effects of WAY100635 on lurasidone-induced 5-HT release mediated by D4 receptor function or not. PD168077 (D4 receptor agonist) alone, and in combination with sub-effective dose lurasidone, increased dopamine release in the mPFC, whereas L745870 (D4 receptor antagonist) did not affect transmitter release in the mPFC on its own or on the ability of lurasidone to increase dopamine release in the mPFC. These results indicate D4 receptor agonism alone is sufficient to increase dopamine release without affecting with the effects of lurasidone on dopamine release in the mPFC [20]. Therefore, lurasidone generates GABAergic disinhibition in the DRN, whereas 5-HT1AR agonism of lurasidone prevents the serotonergic neuronal activation induced by GABAergic disinhibition.

Downregulation or desensitization of 5-HT1AR is considered a major antidepressant mechanism [4,18,22,32]. Various antidepressants, selective serotonin transporter inhibitors, serotonin/norepinephrine transporter inhibitors, and monoamine oxidase inhibitors downregulate or desensitize 5-HT1AR by increasing extracellular 5-HT levels [4,32]. The antidepressant mirtazapine, which is an α2 adrenoceptor antagonist, reportedly did not increase extracellular 5-HT levels acutely, but chronically desensitized 5-HT1AR without increasing extracellular 5-HT levels via 5-HT1AR associated auto-inhibition [18,22,33]. Furthermore, a 5-HT1AR agonist decreased 5-HT release but desensitized 5-HT1AR [34]. These findings indicate that direct activation of 5-HT1AR contributes to 5-HT1AR desensitization. We show that sub-chronic administration of effective doses of lurasidone desensitized 5-HT1AR, probably reflecting partial 5-HT1AR agonism, despite failure to enhance 5-HT release in the DRN (Figure 7). Therefore, based on its receptor binding profile, partial 5-HT1A agonism by lurasidone [13] is consistent with its antidepressive action. 

5-HT1AR exists as functionally two sub-types, autoreceptor and heteroreceptor. Autoreceptors are expressed on the somatodendritic regions of serotonergic neurons in the DRN, which inhibits neuronal activity resulting in reduction of 5-HT release in terminal regions [35]. Contrary, heteroreceptors are expressed on postsynaptic regions in glutamatergic and GABAergic neurons in the cortex and limbic system [35]. The present study chronic lurasidone administration generated autoreceptor desensitization resulting in enhancement of serotonergic transmission from the DRN to mPFC and MDTN. The desensitization of heteroreceptor on the glutamatergic and GABAergic neurons in the mPFC probably activates excitatory glutamatergic and inhibitory GABAergic transmission. The present study demonstrated that the bottom-up regulation (from DRN to mPFC) of lurasidone on serotonergic transmission, whereas the detailed study to determine the top-down regulation (from mPFC to DRN) of lurasidone must be needed.

5-HT2AR antagonism is considered to be one of the major mechanisms of lurasidone (Ki = 2.0 nM) [31], similar to other atypical antipsychotics, such as risperidone, blonanserine, clozapine, zotepine and quetiapine [5,6,10,12]. Previous electrophysiological study demonstrated that 5-HT2AR probably activated both serotonergic and GABAergic neuronal activities in the DRN [19]. Therefore, the effects of 5-HT2AR antagonism on mesocortical and mesothalamic serotonergic transmission is probably more complex rather than those of 5-HT1AR and 5-HT7R, since 5-HT2AR blockade generates GABAergic disinhibition but at the same time prevention of serotonergic activities. We shall the effects of acute and chronic administration of lurasidone mesocortical and mesothalamic serotonergic transmission associated with 5-HT2AR. 

### 3.3. 5-HT7R-Assoicated Mechanisms of Lurasidone

Attenuation of 5-HT7R activities may induce and accelerate antidepressant actions. Recent preclinical studies show that inhibition of 5-HT7R leads to rapid, robust, and sustained antidepressant effects, representing a significant breakthrough in psychopharmacology. Behavioral studies also demonstrated that subeffective doses of SB269970 enhanced the antidepressive actions of several antidepressants [4,16]. Esketamine was approved by the FDA in early 2019 as a rapid-acting antidepressant for the treatment of major depressive disorders [36]. Monoaminergic antidepressants have delayed onset of antidepressive actions and take up to several weeks to exert their salutary effects. These types of antidepressants can improve depressive symptoms, but their ability to successfully reduce suicide ideation and behavior remains inconclusive [37]. In contrast, single doses of ketamine have superior response rates within hours to days of administration [38]. Similarly, single doses (0.5 mg/kg) of intravenous ketamine exert rapid and profound antidepressant effects with reduced suicidal ideation within hours to days of administration [38,39]. Although well-tolerated at sub-anesthetic (antidepressive) doses, ketamine retains its ability to promote abuse and accelerate acute psychotic episodes. 

Various targets have been reported for ketamine, although inhibition of NMDA-R is considered the main pharmacological target [40,41]. Inhibition of NMDA-R activates glutamatergic transmission via GABAergic disinhibition in the cortex and in sub-cortical regions [5,6,9,10,11,12,42,43]. In our hands, MK801 (NMDA-R antagonist) drastically increased 5-HT release via GABAergic disinhibition in the DRN. Conversely, the 5-HT7R antagonists lurasidone and SB269970 induced GABAergic disinhibition, but did not increase 5-HT release in the presence of functional 5-HT1AR. Following inhibition of 5-HT1AR in the DRN, both SB269970 and lurasidone increased 5-HT release in the DRN, MDTN and mPFC. Although both MK801 and 5-HT7R antagonists exhibit rapid-onset antidepressive effects, their actions on 5-HT release differ. These differences between the effects of MK801 and lurasidone on serotonergic transmission suggest that GABAergic disinhibition is a candidate mechanism for rapid-acting antidepressive actions rather than enhancement of serotonergic transmission. 

The pharmacological properties of 5-HT7R have been described [44], but whereas SB269970 acts as a 5-HT7R antagonist, chronic administration of SB269970 desensitizes 5-HT7R and fails to downregulate its expression [44]. We also demonstrate that sub-chronic administration of effective doses of lurasidone desensitized 5-HT7R as shown for SB269970 [44]. Therefore, we suggest that lurasidone is an inverse agonist of 5-HT7R. In agreement, desensitization of 5-HT1AR and 5-HT7R following sub-chronic treatments with lurasidone inhibited their direct and indirect inhibitory (modulated by GABAergic disinhibition) actions on 5-HT release in the DRN, respectively. Accordingly, sub-chronic lurasidone administration enhanced MK801-induced 5-HT release in the DRN, MDTN and mPFC. 

In recent reports, hyperactivation of thalamocortical glutamatergic transmission contributed to the cognitive impairments of several psychotic disorders [9,11,12,42,43,45]. Under physiological conditions, the MDTN regulates various cognitive processes, and enhanced sensitivity and reliability of MDTN signaling contributed to changes in flexibility and stability against environmental changes [45]. Hence, severe GABAergic disinhibition from the reticular thalamic nucleus following attenuation of NMDA-R in the MDTN probably sustains hyperactivity of MDTN glutamatergic activities, resulting in relative deterioration of sensitivity to signals from other regions and leading to functionally similar conditions to those following disruption of MDTN activity [46]. Inhibition of 5-HT7R in the MDTN normalized the hyperactivation of glutamatergic neurons in the MDTN. Although this is a reasonable outcome of antipsychotics, the effects of lurasidone on serotonergic transmission from the DRN to the MDTN remain unknown [11]. We also show that neither subeffective nor effective doses of lurasidone acutely affected 5-HT release in the MDTN. Therefore, taken with previous findings [11], this study suggests that lurasidone normalizes hyperactivation of glutamatergic neuronal hyperactivities in the MDTN by directly inhibiting 5-HT7R in the MDTN without affecting DRN–MDTN serotonergic transmission. Contrary to the known acute effects of lurasidone, chronic administration of lurasidone may activate DRN–MDTN serotonergic transmission, but may also lead to excitatory 5-HT7R desensitization. Alternatively, both acute and chronic treatments with lurasidone could normalize hyperactivated thalamocortical glutamatergic transmission via inverse 5-HT7R agonistic actions. To clarify this hypothesis, further studies of the chronic effects of lurasidone on MK801-induced thalamocortical glutamatergic transmission are awaited. 

## 4. Materials and Methods 

### 4.1. Preparation of the Microdialysis System

All animal care and experimental procedures were performed in compliance with the ethical guidelines established by the Institutional Animal Care and Use Committee at Mie University (No. 29-30). All studies involving animals are reported in accordance with the relevant ARRIVE guidelines [47] and European Union Council (2010/63/EU). A total of 132 rats were used in experiments.

Male Sprague-Dawley rats (approximately 250 g, 7−8 weeks old, SLC, Shizuoka, Japan) were maintained in a controlled environment (22 °C ± 1 °C) with a 12-h light/12-h dark cycle. Especially, in Study_6, male Sprague-Dawley rats (approximately 200 g, 7 weeks old, SLC) were administrated lurasidone (3 mg/kg/day for 7 days) by subcutaneously osmotic pump (2ML_1, Alzet, Cupertino, CA). The nominal pumping rate and duration of 2ML_1 osmotic pump are 10 μL/h and 7 days, respectively. All rats were weighed before the study. Rats were anesthetized with 1.8% isoflurane and were then placed in a stereotaxic frame for implantation of dialysis probes [5,7]. Concentric direct insertion-type dialysis probes (0.22 mm diameter; Eicom, Kyoto, Japan) were implanted in the mPFC (3 mm exposed membrane; A = +3.2 mm, L = +0.8 mm, V = −5.2 mm, relative to the bregma), MDTN (2 mm exposed membrane; A = −3.0 mm, L = +0.9 mm, V = −6.2 mm, relative to the bregma at a lateral angle of 30°) and DRN (1 mm exposed membrane; A = −8.2 mm, L = 0.2 mm, V = −6.8 mm, relative to the bregma at a lateral angle of 15°) [48]. During recovery and experimentation, rats were housed individually in cages and were provided food and water *ad libitum*. Perfusion experiments were initiated at 18 h after recovery from isoflurane anesthesia [8,49,50]. During experiments, single rats were placed in an in vivo dialysis system for freely moving animals (Eicom) equipped with a two-channel swivel (TCS2-23; ALS, Tokyo, Japan). The perfusion rate was set at 2 μL/min in all experiments using modified Ringer’s solution (MRS, composition described below) [7,50], and dialysates were collected over 20 min sampling epochs. Extracellular l-glutamate levels were measured at 8 h after the start of perfusions. After baseline recording, perfusion medium was switched to MRS containing MK801, WAY100635, BP554, AS19, muscimol, SB269970 or lurasidone as indicated. Dialysate samples were then injected into the UHPLC apparatus. All samples were taken from freely moving animals.

After microdialysis experiments, brains were removed following cervical dislocation and overdose isoflurane anesthesia. The locations of the dialysis probes were verified in each animal using histological examinations of 200 μm thick brain tissue slices, which were prepared using a Vibratome 1000 (Technical Products International Inc., St. Louis, MO).

### 4.2. Determination of Extracellular GABA and 5-HT Levels

GABA concentrations in the perfusate (μM) were determined using UHPLC (xLC3185PU; Jasco) with fluorescence resonance energy transfer detection (xLC3120FP; Jasco, Tokyo, Japan) after dual derivatization with isobutyryl-l-cysteine and *o*-phthalaldehyde. Derivative reagent solutions were prepared by dissolving isobutyryl-l-cysteine (2 mg) or *o*-phthalaldehyde (1 mg) in 0.1 mL aliquots of ethanol, followed by addition of 0.9 mL of sodium borate buffer (0.2 M, pH 9.0) [50]. Automated precolumn derivation was conducted by mixing 5 μL sample, standard, or blank solutions with 5 μL of derivative reagent solution in reaction vials for 5 min before injection. Derivative samples (5 μL) were injected using an autosampler (xLC3059AS; Jasco). The analytical column (YMC Triart C18, particle 1.8 μm, 50 × 2.1 mm; YMC, Kyoto, Japan) was maintained at 45 °C. The flow rate was set at 500 μL/min, and elution was performed using a linear gradient of mobile phases A (0.05 M acetate buffer, pH 5.0) and B (0.05 M acetate buffer containing 60% acetonitrile, pH 3.5) over 10 min [51]. Excitation and emission wavelengths of the fluorescence detector were set at 280 and 455 nm, respectively.

Concentrations of 5-HT in the perfusate (nM) were determined using UHPLC (xLC3185PU; Jasco) with electrochemical detection (ECD-300; Eicom, Kyoto, Japan) by a graphite carbon electrode set at +450 mV (vs. a Ag/AgCl reference electrode [49,52]. The analytical column (Triart C18, particle 1.8 μm, 30 × 2.1 mm; YMC) was maintained at 40 °C and the flow rate of the mobile phase was set at 400 μL/min. The mobile phase contained 0.1 M acetate buffer, 1% methanol, and 50 mg/L EDTA–2Na (final pH 6.0) [53,54]. Where possible, we randomized and blinded sample data. In particular, for determinations of extracellular transmitter levels, the sample order was dictated by the autosampler according to a random number table.

### 4.3. Data Analysis

Where possible, we randomized and blinded sample data. To determine extracellular transmitter levels, the sample order was set on the autosampler according to a random number table. Drug doses and sample sizes were selected according to previous studies [11,42,45]. All experiments in this study were designed with equally sized animal groups (N = 6) [9,11,42,43,45] and all values were expressed as means ± standard deviations (SD). Differences were considered significant when P < 0.05 (two-tailed).

Regional transmitter concentrations were analyzed using Mauchly’s sphericity test followed by multivariate analysis of variance (MANOVA) using BellCurve for Excel ver. 3.20 (Social Survey Research Information Co., Ltd., Tokyo, Japan). The data composed of average of pre-treatment period (1 point) and each time point during target agent administration (9 points). When the data did not violate the assumption of sphericity (P > 0.05), the F-value of MANOVA was analyzed using sphericity-assumed degrees of freedom. When the assumption of sphericity was violated (P < 0.05), F-values were analyzed using Chi–Muller’s corrected degrees of freedom by BellCurve for Excel. When F-values for drug factors were significant in MANOVA, the data were finally analyzed using Tukey’s post hoc test with BellCurve for Excel. Transmitter levels were expressed as the area under the curve between 20 and 180 min (AUC20–180 min) after target agents administration. All statistical analyses complied with the recommendations for experimental design and analysis in pharmacology [55].

### 4.4. Chemical Agents

Lurasidone, the 5-HT1AR agonist BP554, the 5-HT7R antagonist SB269970, the GABA_A_-R agonist muscimol, and the noncompetitive NMDA-R antagonist MK801 were obtained from Fujifilm-Wako (Osaka, Japan). The 5-HT1AR antagonist WAY100635 and the 5-HT7R agonist AS19 were purchased from Cosmo-Bio (Tokyo, Japan).

All compounds were prepared on the day of experiments. All drugs were perfused in MRS containing 145 mM Na^+^, 2.7 mM K^+^, 1.2 mM Ca^2+^, 1.0 mM Mg^2+^, and 154.4 mM Cl^−^, which was adjusted to pH 7.4 using 2 mM phosphate buffer and 1.1 mM Tris buffer [7,50]. Muscimol, MK-801, WAY100635, and SB269970 were dissolved in MRS directly, whereas BP554 and AS19 were initially dissolved in dimethyl sulfoxide at 50 mM. Lurasidone was initially dissolved in dimethyl sulfoxide at 1 mg/mL. The final dimethyl sulfoxide concentration was lower than 0.1% (vol/vol).

## 5. Conclusions

In the present study, we demonstrated interactions between 5-HT1AR and 5-HT7R that are relevant to serotonergic and GABAergic transmission and explored the mechanisms behind the antipsychotic and mood stabilizing actions of lurasidone. Serotonergic and GABAergic neuronal activities in the DRN were predominantly regulated by inhibitory 5-HT1AR and excitatory 5-HT7R responses, respectively. 5-HT7R antagonism of lurasidone generates GABAergic disinhibition, but partial agonism of 5-HT1AR by lurasidone prevents 5-HT release, resulting in no net changes in basal 5-HT release. Failure to increase 5-HT release in the MDTN and the mPFC reflects the interaction between 5-HT1AR partial agonism and 5-HT7R antagonism by lurasidone. Furthermore, 5-HT7R antagonism inhibits NMDA-R-antagonist induced thalamocortical glutamatergic transmission, but enhances serotonergic transmission via GABAergic disinhibition in the DRN. Finally, lurasidone acutely inhibits 5-HT release following NMDA-R inhibition by acting as a 5-HT1AR agonist, but chronically activates NMDA-R antagonist induced serotonergic transmission by desensitizing 5-HT1AR and 5-HT7R. These interactions between 5-HT1AR agonism and 5-HT7R antagonism of lurasidone probably contribute to the preclinically observed rapid-action and augmentation of antidepressive actions by lurasidone.

## Figures and Tables

**Table 1:** Summary of study designs.

	Administration Root (Target Agent)	TARGET AGENT	Pre-Treatment Roots	Pre-Treatment Agents	Reference
Study_1	Acute systemic (i.p.)	LUR (0.3 and 1 mg/kg)	-	-	[11,17,20]
Study_2	Acute systemic (i.p.)	LUR (0.3 and 1 mg/kg)	Local (DRN)	WAY (10 μM)	[11,17,20]
Study_3	Local (DRN)	LUR (1 and 3 μM) BP (50 μM) AS (1 μM) SB (10 μM) Muscimol (1 μM)	-	-	[11] [8] [21] [11] [6]
Study_4	Local (DRN)	LUR (1 and 3 μM) AS (1 μM) SB (10 μM)	Local (DRN)	WAY (10 μM)	[11,22] [21] [11]
Study_5	Local (DRN)	MK801 (5 μM)	Local (DRN)	LUR (3 μM) LUR (3 μM) + WAY (10 μM) BP (50 μM) SB (10 μM) Muscimol (1 μM)	[11,12] [11] [8] [11] [6]
Study_6	Local (DRN)	MK801 (5 μM) BP (50 μM) AS (1 μM)	Chronic systemic (s.c.)	LUR (3mg/kg/day) for 7 days	[12] [17] [20]

Lurasidone (LUR), N-[2-[4-(2-Methoxyphenyl)-1-piperazinyl]ethyl]-N-2-pyridinylcyclohexanecarboxamide (WAY: 5-HT1AR antagonist), 1-[3-(3,4-Methylenedioxyphenoxy)propyl]-4-phenyl-piperazine (BP: 5-HT1AR agonist), (2S)-(+)-5-(1,3,5-Trimethylpyrazol-4-yl)-2-(dimethylamino)tetralin (AS: 5-HT7R agonist), (2R)-1-[(3-Hydroxyphenyl)sulfonyl]-2-[2-(4-methyl-1-piperidinyl)ethyl]pyrrolidine (SB: 5-HT7R antagonist), Muscimol (MUS: GABA_A_-R agonist).
